# Minimal dose of milk protein concentrate to enhance the anabolic signalling response to a single bout of resistance exercise; a randomised controlled trial

**DOI:** 10.1186/s12970-017-0175-x

**Published:** 2017-06-08

**Authors:** Cameron J. Mitchell, Nina Zeng, Randall F. D’Souza, Sarah M. Mitchell, Kirsten Aasen, Aaron C. Fanning, Sally D. Poppitt, David Cameron-Smith

**Affiliations:** 1 Liggins Institute, The University of Auckland, Private Bag 92 019, Victoria Street West, Auckland, 1142 New Zealand; 2 School of Biological Sciences, The University of Auckland, Private Bag 92 019, Victoria Street West, Auckland, 1142 New Zealand; 3 Fonterra Research and Development Centre, Private Bag 11 029, Palmerston North, 4442 New Zealand

**Keywords:** Skeletal muscle, Middle age, P70SK, Supplement, Protein phosphorylation

## Abstract

**Background:**

Resistance training is a potent stimulus to induce muscle hypertrophy. Supplemental protein intake is known to enhance gains in muscle mass through activation of the mammalian target of rapamycin complex 1 (mTORC1) pathway, which initiates protein translation. While the optimal dose of high quality protein to promote post exercise anabolism in young or older men has been investigated, little is known about the minimum doses of protein required to potentiate the resistance exercise activation of anabolic signalling in middle aged men.

**Methods:**

Twenty healthy men (46.3 ± 5.7 years, BMI: 23.9 ± 6.6 kg/m^2^) completed a single bout of unilateral resistance exercise consisting of 4 sets of leg extension and press at 80% of 1 repetition maximum. Participants were randomised to consume either formulated milk product containing 9 g milk protein (FMP) or an isoenergetic carbohydrate placebo (CHO) immediately post exercise, in a double blind fashion. A single muscle biopsy was collected at pre-exercise baseline and then bilateral biopsies were collected 90 and 240 min after beverage consumption.

**Results:**

P70S6K^Thr389^ phosphorylation was increased with exercise irrespective of group, P70S6K^Thr421/Ser424^ was increased with exercise only in the FMP group at 240 min. Likewise, rpS6 ^Ser235/236^ phosphorylation was increased with exercise irrespective of group, rpS6 ^Ser240/244^ increased to a greater extent following exercise in the FMP group. mRNA expression of the amino acid transporter, LAT1/ *SLC7A5* increased with both exercise and beverage consumption irrespective of group. PAT1/ *SLC36A1*, CAT1/ *SLC7A1* and SNAT2/ *SLC38A2* mRNA increased only after exercise regardless of group.

**Conclusions:**

Nine grams of milk protein is sufficient to augment some measures of downstream mTORC1 signalling after resistance exercise but does not potentiate exercise induced increases in amino acid transporter expression. Formulated products containing nine grams of milk protein would be expected stimulate muscle anabolism after resistance exercise.

**Trial registration:**

New Zealand Clinical Trials Registry ACTRN12615001375549. Registered: 17 December, 2015.

## Background

Skeletal muscle mass and function are key determinants of both metabolic health [1] and physical performance [2, 3]. Importantly for an ageing population, muscle mass and function is reduced with increasing age, impairing whole body insulin sensitivity [1] and mobility [4]. There is a measurable decline in muscle mass commencing from around the fourth decade of life [5], hence middle age is an obvious target for interventions to preserve muscle mass. Yet despite this, few studies have examined effective strategies for this demographic.

While resistance training can increase muscle mass and strength in both young and older adults [6], the relative magnitude of these gains decrease with age [7–9]. Post-exercise protein intake has been shown to augment resistance training induced muscle mass gains [10]. In older adults greater protein doses are required to maximally stimulate muscle protein synthesis (MPS) [11] and the necessary anabolic signalling within muscles [12]. Yet despite the importance of establishing a maximal effective dose, it is also necessary to establish the efficacy of smaller protein doses, including those that are typically incorporated into a range of consumer goods. The addition of large amounts of protein to mass market products can increase the cost to the consumer, alter taste [13] and limit formulation options [14]. Thus many foods consumed after resistance exercise may include a suboptimal protein dose.

Muscle mass is regulated by both anabolic and catabolic processes [15]. Following resistance exercise and protein ingestion, there is a much greater magnitude increase in anabolic processes compared to the suppression of catabolism [16], thus anabolic processes are thought to exert a primary regulatory role in dictating muscle mass gains [17, 18]. The increase in MPS in response to both exercise and feeding is driven by the initiation of protein translation [19]. This occurs via the pathway centred on the mammalian target of rapamycin complex 1 (mTORC1), which integrates signals from growth factors, mechanical stimuli, cellular energy status and amino acids. Once activated, mTORC1 phosphorylates a number of downstream effectors including P70S6 kinase (P70S6K) and ribosomal protein S6 (rpS6) which subsequently enable the initiation of protein translation [20]. Studies have correlated the activation of these kinases with both acute increases in MPS [21] and long term gains in muscle size [22, 23].

Previously it has been demonstrated that high dose milk protein concentrate (MPC) consumption results in an equivalent anabolic response to a matched dose of whey protein, in middle-aged men at rest [24]. Further studies from our group have also shown that as little as 10 g dose of MPC is sufficient to stimulate MPS with no effect on downstream anabolic signalling in this group [25]. Thus, the purpose of the present study was to determine if a similar quantity of milk protein would augment the anabolic signalling response downstream of mTORC1, to a single bout of resistance exercise in middle-aged men. Further analyses were undertaken to characterize the transcriptional response of skeletal muscle amino acids transporters LAT1/ *SLC7A5,* PAT1/, *SLC36A1*, CAT1/ *SLC7A1* and SNAT2/ *SLC38A2* which have been shown to increase in response to resistance exercise [26] and large doses of essential amino acids [27].

## Methods

### Participants

Twenty men between the ages of 38 and 55 years participated in the study. The men were not taking any medication and had no neuromuscular, metabolic or cardiovascular medical conditions. Participants were sedentary to recreationally active, but did not participate in resistance training. Baseline characteristics are shown in Table 1. All participants provided informed verbal and written consent, the study was approved by the Health and Disability Ethics Committee (New Zealand, Ref# 15/NTB/154/AM01) and registered with the Australia New Zealand Clinical Trials Registry ACTRN12615001375549.

**Table 1:** Subject characteristics

	Placebo (*n* = 10)	9 g MPC (*n* = 10)
Age (years)	45.5 ± 5.8	48.6 ± 4.6
Height (cm)	175.9 ± 5.6	179.5 ± 6.1
Weight (kg)	79.3 ± 9.1	76.3 ± 8.4
BMI (kg/m^2^)	24.6 ± 2.4	24.4 ± 2.8
% Body fat	19.3 ± 7.6	18.2 ± 5.7
HDL cholesterol (mmol/L)	1.36 ± 0.30	1.52 ± 0.59
LDL cholesterol (mmol/L)	4.13 ± 1.10	3.37 ± 0.98
Triglycerides (mmol/L)	1.27 ± 0.52	1.24 ± 0.55
HOMA-IR	1.81 ± 1.33	1.18 ± 0.34
Leg press 1RM (kg)	89.9 ± 20.3	92.5 ± 23.8
Leg extension 1RM (kg)	46.9 ± 11.2	45.3 ± 15.7

Body mass index (BMI), high-density lipoprotein (HDL), low-density lipoprotein (LDL), Homeostatic model assessment of insulin resistance (HOMA-IR), one repetition maximum (1RM). Data are shown as mean ± SD

### Study beverages

Using a parallel double blind design, participants were randomized to consume one of two study beverages, using sequences generated with www.random.org. Beverages were Functional milk product (FMP) (9 grams of protien; milk protein; Fonterra Co-operative Group Ltd, Auckland, New Zealand) or an isoenergetic carbohydrate placebo (CHO). The FMP was manufactured from spray dried skim milk powder, spray dried milk protein concentrate, and additional calcium, magnesium, zinc, and vitamin C. The macronutrient and essential amino acid composition are shown in Table 2. Powders were dissolved in 250 ml of water. Total protein in the beverages was calculated as 6.38 multiplied by total nitrogen determined using the Kjeldahl method. Essential amino acid composition of the FMP was determined using high-performance liquid chromatography.

**Table 2:** Study beverage composition

	Functional milk product	Carbohydrate placebo
Energy (kJ)	504	491
Carbohydrate (g)	13.8	28.0
Fat (g)	3.2	0.6
Protein (g)	9.2	0.1
Essential amino acids (g)	4.0	-
Threonine (g)	0.43	-
Valine (g)	0.53	-
Isoleucine (g)	0.46	-
Leucine (g)	0.87	-
Phenylalanine (g)	0.42	-
Lysine(g)	0.72	-
Histidine (g)	0.23	-
Methionine (g)	0.21	-

### Experimental design

At least 3 days prior to their clinical visit participants were familiarized with the leg press and leg extension apparatus. One repetition maximum (1RM) for each exercise was estimated using the Brzycki [28] equation based on a repetition set where muscle failure was achieved in less than 6 repetitions. Participants also underwent a full body dual energy x-ray absorptiometry (DXA, Lunar Prodigy, GE, Waltham, MA, USA) scan to quantify lean, fat and total mass. The day prior to the study visit participants consumed a standard evening meal containing 670 kcal (50% CHO, 25% protein, 25% fat). All participants arrived at the laboratory in the fasted state. Upon arrival, a cannula was inserted into an antecubital vein, a pre-exercise baseline blood sample was collected and a saline drip was then maintained for the remainder of the trial to keep the cannula patent. A muscle biopsy was obtained from the *vastus lateralis* of a random leg (counterbalanced for dominance) with a bergstrom needle modified for manual suction, under local anaesthesia (1% lidocaine).

Participants then completed four sets of unilateral leg press and extension using the leg contralateral to the first biopsy. Each set was performed at 80% of the participant’s estimated 1RM, 10 repetitions were completed for the first three sets and the last set of each exercise was completed to the point of momentary muscular failure. After the completion of the exercise, a post exercise (0 min) blood sample was collected and participants then consumed their assigned study beverage in full within three minutes. Blood samples were then obtained in ethylenediaminetetraacetic acid coated vacutainers at 30, 60, 90, 120, 180 and 240 min after the study beverage was consumed.

Bilateral muscle biopsies were obtained 90 and 240 min after the beverage was consumed. This study design allowed for the delineation effects of exercise and protein feeding alone (FED) and together (FED-EX).

### Plasma measurements

Baseline blood biochemistry and the time course of plasma glucose levels were analysed using a Roche C311 autoanalyzer, (Roche, Mannheim, Germany) by enzymatic colorimetric assay. Plasma insulin concentrations were analysed using electrochemiluminescence immunoassay on Cobas e 411 (Roche, Mannheim, Germany). The homeostatic model assessment (HOMA) insulin resistance was calculated based as reported by Mathews et al. [29]. Plasma samples were deproteinised by tungstate precipitation and amino acid concentrations were measured using a fluorescent derivitisation utilising the reaction of amino nitrogen with 6-aminoquinolyl-N-hydroxysuccinimidyl carbonate and subsequent separation by ultra-high pressure liquid chromatography as previously described [30].

### Immunoblotting

Protein extractions were conducted as per Mitchell et al [24]. Briefly, muscle biopsies (40–50 mg) were homogenised for 40 s at 20 Hz using a TissueLyser (Qiagen, Venlo, Netherlands) in RIPA buffer (10 μL/mg 25 mM Tris 0.5% v/v Triton X-100) and protease/phosphatase inhibitor cocktail (HaltTM Protease and Phosphatase Inhibitor Cocktail, Thermo Scientific, cat. 78442). Samples were then centrifuged at 4500 g for 10 min at 4 °C. The supernatant was collected for western blot analysis. The total protein content was determined using a BCA-protein kit following the manufacturer’s protocols (Thermo Fisher Scientific, Waltham, MA, USA). Aliquots of 20 μg total protein were prepared, suspended in Laemmli buffer, boiled, and subjected to SDS-PAGE. Western blotting was performed as previously described [12] using sodium dodecyl sulphate-polyacrylamide gel electrophoresis then transferred to a polyvinyl fluoride membrane using the Trans-Blot Turbo™ Transfer System (Bio-Rad, Hong Kong, China). membranes were then blocked in 5% BSA/Tris Buffer Saline/0.1% Tween 20 (TBST) for 2 h (h) at room temperature, followed by overnight incubation at 4 °C with gentle agitation with primary antibodies (1:1000) (p-P70S6K (Thr389) (Cell Signalling, 9206S), p-P70S6K (Thr421/Ser424) (Cell Signalling, 9204S), p-rps6 (Ser235/236) (Cell Signalling, 4865S), p-rps6 (Ser240/244) (Cell Signalling, 2215S), p-ERK1/2 (Thr202/Tyr204) (Cell Signalling, 4370S), p-P90RSK (Ser380) (Cell Signalling, 11989S), LAT1 (Cell Signalling, 5347S), SNAT2 (Santa Cruz, 67081), CAT1 (Abcam, ab37588) and GAPDH (Abcam, ab9485)). The following morning the membranes were washed for 30 min with TBST and probed with HRP conjugated goat anti-rabbit or goat anti-mouse secondary antibodies (Jackson ImmunoResearch, West Grove, PA, USA) for 1 h at room temperature. Following 30 min further washing in TBST, antibody binding was visualized using ECL Select Western blotting detection reagent (Amersham, UK) and chemiluminescent signals were captured using a ChemiDoc™ MP Imaging System (Bio-Rad, Hong Kong, China). Densitometry analysis of protein bands was performed using Image J software. Abundance of proteins of interest was normalized for protein loading by stripping and re-probing membranes for GAPDH.

### Real-time polymerase chain reaction

Total RNA was extracted from ~20 mg of muscle tissue using the AllPrep^®^ DNA/RNA/miRNA Universal Kit (QIAGEN GmbH, Hilden, Germany) following the manufacturer's instructions. 1500 ng of input RNA was used for cDNA synthesis using High‐Capacity RNA‐to‐cDNA^™^ kit (Life Technologies, Carlsbad, CA), messenger RNA (mRNA) were measured by Real-time polymerase chain reaction on a LightCycler 480 II (Roche Applied Science, Penzberg, Germany) using SYBR Green I Master Mix (Roche Applied Science). As per Figueiredo et al. [31] target mRNAs were LAT1/*SLC7A5*, PAT1/*SLC36A1*, CAT1/*SLC7A1*, and SNAT2/*SLC38A2*. Primers were designed using BLAST [32] software with sequences in Table 3. The geometric mean of five reference genes [33] was used for normalisation. The recently proposed human reference genes [34], ER membrane protein complex subunit 7 (*EMC7*), valosin-containing protein (*VCP*), charged multivesicular body protein 2A (*CHMP2A),* chromosome 1 open reading frame 43 (*C1orf43*) and hypoxanthine phosphoribosyltransferase 1 (*HRPT*) were identified as the least variable and therefore, used as reference genes (Table 4). Standard and melting curves were performed for every target to confirm primer efficiency and single‐product amplification. Due to insufficient sample volumes, RNA data at 90 min post beverage consumption was not available.

**Table 3:** Primer sequences

LAT1 (Forward)	CATCCTCCAGGCTCTTCTTCGTG
LAT1 (Reverse)	AAGGCGTAGAGCAGCGTCAT
SNAT2 (Forward)	GCAGTGGAATCCTTGGGCTT
SNAT2 (Reverse)	AAAGACCCTCCTTCATTGGCAG
CAT1 (Forward)	TCTCATTTAAGGTTCCCTTCCTG
CAT1 (Reverse)	ACAGGCCATAGCCAAAGTAGA
PAT1 (Forward)	CTTCTGCCGCAGGCTGAATAAA
PAT1 (Reverse)	CAGGAAGAAGTCCACAACACG
SESTRIN1 (Forward)	TTTCGTGTCCAGGACTATTGC
SESTRIN1 (Reverse)	ACTGTCCCACATCTGGATAAAGG
SESTRIN2 (Forward)	CAACCTCTTCTGGAGGCACTT
SESTRIN2 (Reverse)	CCTGCTCAGGAGTCAGGTCA
SESTRIN3 (Forward)	CAGGCAGCAACTTTGGGATTGT
SESTRIN3 (Reverse)	AGACGCCTCTTCATCTTCCCTT

**Table 4:** Primer sequences for housekeeping genes

Gene	Sequence
EMC7 (Forward)	GGGCTGGACAGACTTTCTAATG
EMC7 (Reverse)	CTCCATTTCCCGTCTCATGTCAG
VCP (Forward)	AAACTCATGGCGAGGTGGAG
VCP (Reverse)	TGTCAAAGCGACCAAATCGC
CHMP2A (Forward)	CGCTATGTGCGCAAGTTTGT
CHMP2A (Reverse)	GGGGCAACTTCAGCTGTCTG
C1orf43 (Forward)	CTATGGGACAGGGGTCTTTGG
C1orf43 (Reverse)	TTTGGCTGCTGACTGGTGAT
HPRT (Forward)	CCTGGCGTCGTGATTAGTGAT
HPRT (Reverse)	TCGAGCAAGACGTTCAGTCC

### Statistical analysis

Differences in protein phosphorylation and gene expression were tested using three-way ANOVA with time and exercise as repeated fixed factors and study beverage as a between subject fixed factor. Differences in plasma parameters were tested with two-way ANOVA with time as a repeated fixed factor and study beverage as a between subject fixed factor. Sidak *post hoc* test was used when significant interactions were present. All analysis was conducted using SPSS version 23 (IBM, Armonk, New York, United States). Alpha was set at *P* < 0.05 and data are shown as means ± standard deviation (SD) in the tables and text and ± standard error of the mean (SEM) in the figures.

## Results

### Plasma

**Fig. 1:**
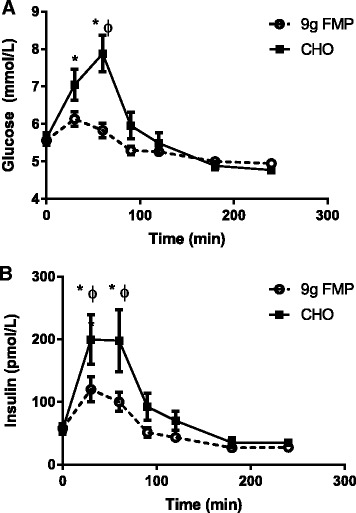
Glucose and Insulin. Plasma concentrations of glucose (**a**) and insulin (**b**) following study beverage ingestion. The dashed line with open circles represents the FMP group and the solid lines with solid squares represent the CHO group. Error bars represent SEM. * = significantly different from time 0 with in the same group (*P* < 0.05). Φ = significantly different between groups at indicated time point (*P* < 0.05)

**Fig. 2:**
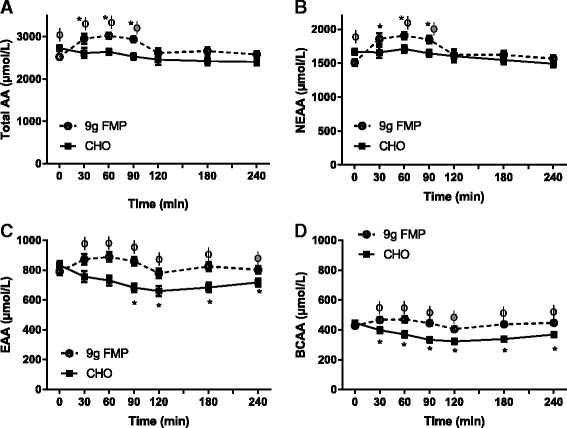
Amino acids. Plasma concentrations of amino acids (AA) (**a**), non-essential amino acids (NEAA) (**b**), essential amino acids (EAA) (**c**), and branched chain amino acids (BCAA) (**d**) following study beverage ingestion. The dashed line with open circles represent the FMP group and the solid lines with solid squares represent the CHO group. Error bars represent SEM. * = significantly different from time 0 with in the same group (*P* < 0.05). Φ = significantly different between groups at indicated time point (*P* < 0.05)

As expected CHO ingestion increased both glucose (time X group *P* < 0.001) and insulin (time X group *P* = 0.015) concentrations above baseline and were greater than the concentrations observed for FMP 30 and 60 min after ingestion of the beverage (*P* < 0.05) (Fig. 1a&b). Total amino acid concentrations (Fig. 2a) (time X group *P* = 0.016) were elevated above baseline at 30, 60 and 90 min after the ingestion of 9 g of FMP (*P* < 0.05) but were not altered in the CHO group. There was also a difference between the two groups over this time period (*P* < 0.05). Non-essential amino acids (Fig. 2b) followed the same pattern (time X group *P* = 0.057, group *P* = 0.012, time *P* < 0.001) and were elevated only after FMP consumption at 30 and 90 min (*P* < 0.05). There was also a difference between groups over this time period (*P* < 0.05). Essential amino acid concentrations (Fig. 2c) (time X group *P* = 0.012) were greater in the FMP group compared to the CHO group for the entire post beverage ingestion period (*P* < 0.05). Essential amino acid concentrations were depressed below baseline from 90 min post beverage ingestion for the remainder of the measurement period only in the CHO group. Branched chain amino acids (Fig. 2d) (time X group *P* = 0.010) were depressed for the full measurement period after study beverage consumption only in the CHO group (*P* < 0.05), there were also differences between groups for the full post beverage period (*P* < 0.05).

### Anabolic signalling

**Fig. 3:**
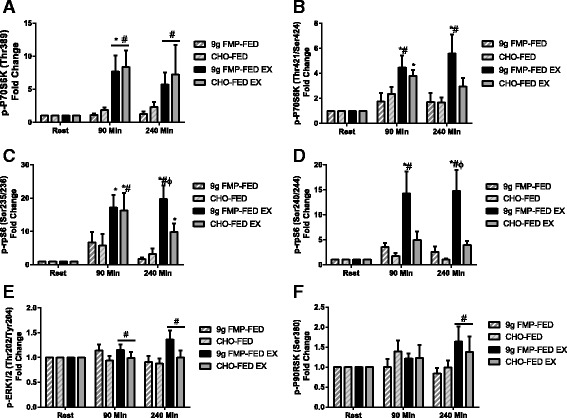
Anabolic signalling. Fold changes in phosphorylation status of P70S6K^Thr389^ (**a**), P70S6K^Thr421/Ser42^4 (**b**), rpS6 ^Ser235/236^ (**c**), rpS6 ^Ser240/244^ (**d**), ERK1/2^Thr202/Tyr204^ (**e**) and P90RSK^Ser360^ (**f**) after feeding both at rest and following resistance exercise. Error bars represent SEM, horizontal line represent a main effect. # = significantly different from FED only leg at same time point (*P* < 0.05). Φ = significantly different between groups at indicated time point, within the exercise condition (*P* < 0.05). * = significantly different from rested baseline samples (*P* < 0.05)

Phosphorylation of P70S6K at the threonine 389 residue (leg X time *P* = 0.001) increased with exercise 90 min after beverage consumption (*P* = 0.004). Phosphorylation of the FED-EX leg was also greater than the FED leg at 90 and 240 min post beverage consumption (*P* < 0.05). There was no difference between groups (group X time *P* = 0.830) (Fig. 3a). P70S6K phosphorylation at the threonine 421/serine424 site increased above baseline (leg X time *P* = 0.001, group X leg *P* = 0.117, time X leg X group *P* = 0.257) in the exercise leg of both groups 90 min post beverage consumption (FMP *P* = 0.002, CHO *P* = 0.022), phosphorylation was greater in the exercise leg than the rest leg at this time point only in the FMP group (*P* = 0.004). At 240 min after beverage consumption, phosphorylation was increased only in the exercise leg of the FMP group (*P* = 0.005), and was greater in the exercise leg of the FMP group than the rest leg at the same time (*P* = 0.001) (Fig. 3b). rpS6 phosphorylation at the serine235/236 site increased in the exercise leg of both groups at both 90 and 240 min after beverage consumption (leg X time *P* = 0.010, group X leg *P* = 0.177, time X leg X group *P* = 0.256), with greater phosphorylation observed in the exercise than the rest leg at 90 min in the CHO group (*P* = 0.049) and at 240 min in the FMP group (*P* < 0.001), and phosphorylation in the exercise leg at 240 min was greater following FMP than CHO (*P* = 0.048) (Fig. 3c). Phosphorylation of rpS6 at the serine240/244 site (leg X time *P* = 0.001, group X leg *P* = 0.007, time X leg X group *P* = 0.076), increased above baseline with exercise in the FMP group at both 90 and 240 min after beverage consumption (*P* < 0.001) and was greater than in the resting leg (*P* = 0.010). rpS6 phosphorylation was greater in the exercise leg of the FMP group compare to the CHO group at 240 min post beverage consumption (*P* = 0.022) (Fig. 3d). Phosphorylation of ERK1/2, (leg *P* = 0.041, leg X time *P* = 0.061) was increased with exercise regardless of time point and with no difference between groups (Fig. 3d). p90RSK phosphorylation at serine380 (leg X time *P* = 0.034) was increased with exercise at 240 min post beverage consumption (*P* = 0.006) when compared to the FED only leg (Fig. 3e).

### Amino acid transporters

**Fig. 4:**
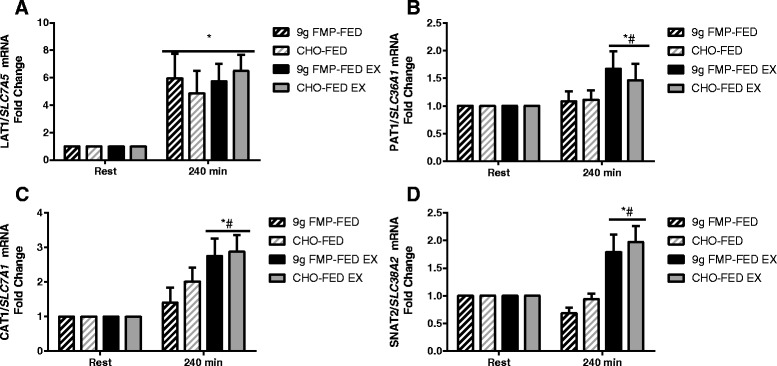
Amino acid transporter gene expression. The mRNA expression of LAT1/ *SLC7A5* (**a**), PAT1/ *SLC36A1* (**b**), CAT1/ *SLC7A1* (**c**) and SNAT2/ *SLC38A2* (**d**). Data are expressed as fold change from rest, error bars represent SEM. Horizontal lines represent a main effect# = significantly different from FED only leg at same time point (*P* < 0.05). * = significantly different from rested baseline samples (*P* < 0.05)

mRNA expression of LAT1/ *SLC7A5* increased 240 min after study beverage ingestion (*P* < 0.001) regardless of exercise or the beverage consumed (Fig. 4a). PAT1/ *SLC36A1 (*time X leg *P* = 0.027), CAT1/ *SLC7A1 (*time X leg *P* = 0.029), and SNAT2/ *SLC38A2*mRNA *(*time X leg *P* < 0.001), expression increased 240 min after beverage consumption only in the exercise leg and was greater than expression in the FED only leg (Fig. 4b, c & d).

**Fig. 5:**
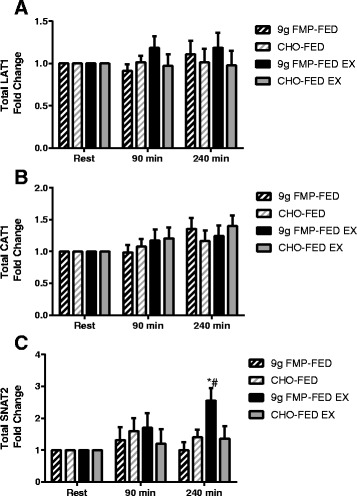
Amino acid transporter protein expression. The protein expression of LAT1 (**a**), CAT1 (**b**), and SNAT2 (**c**). Data are expressed as fold change from rest, error bars represent SEM. # = significantly different from FED only leg at same time point (*P* < 0.05). * = significantly different from rested baseline samples (*P* < 0.05)

**Fig. 6:**
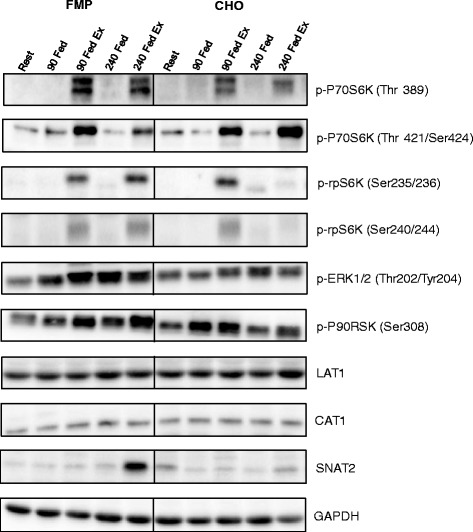
Representative images of western blots for data shown in Figs. 3 and 5

Protein expression of LAT1 and CAT1 was unaltered by beverage consumption or exercise (Fig. 5a&b). SNAT2 protein expression (time X leg X group *P* = 0.021) was increased 240 min after beverage consumption only in the exercise leg of the FMP group (*P* = 0.026) and was greater than the FED only leg at the same time point (*P* = 0.005) (Fig. 5c). Representative images of western blots are shown in Fig. 6.

## Discussion

Resistance exercise is a potent stimulator of muscle hypertrophy [35], MPS [36] and downstream mTORC1 signalling [12]. Protein feeding acts synergistically with resistance exercise to augment the signalling and MPS response observed with resistance training alone [12, 37]. Although less clear for muscle hypertrophy, the dose of protein required to maximally stimulate MPS and anabolic signalling after resistance exercise has been established and is known to increase with age [38]. The present study established that in middle-aged males (46.3 ± 5.7 years) 9 grams of milk protein augments the signalling response to resistance exercise. Yet this dose of protein did not impact gene expression of major skeletal muscle amino acid transporters when consumed immediately post exercise. Therefore, the augmentation of post exercise mTORC1 signalling by 9 grams of milk protein is likely to enhance muscle anabolism even though the magnitude of this potential effect is difficult to quantify based on the current data.

The two beverages used in the present study differed in protein content but were isoenergetic, with the balance being made up by CHO. The CHO placebo stimulated a much larger insulin and glucose response when compared to the protein beverage. Although, the greater insulin response provoked by the CHO may have stimulated signalling upstream of mTORC1, consistent with previous studies, insulin alone was insufficient to stimulate downstream signalling or MPS [25, 39].

Both total and non-essential amino acid concentrations were unaltered by CHO ingestion combined with unilateral resistance exercise, whilst the ingestion of 9 grams of FMP resulted in a transient increase in these concentrations. Conversely, essential and branched chain amino acid concentrations were depressed by CHO ingestion combined with unilateral exercise whereas ingestion of the FMP maintained essential and branched chain amino acid concentrations for the duration of the study period. A small suppression of plasma essential amino acid concentrations following resistance exercise in the fasted state has previously been reported [37, 40] and may be the result of increased catabolism of branched chain amino acids [41]. It is not clear if the observed suppression of essential amino acids is the result of increased uptake promoted by the exercise or due to insulin which was secreted in response to the CHO placebo. Based on classic work by Biolo et al. [42] it appears unlikely that the decreased plasma essential amino acid concentration was the result of uptake by the exercised muscle and therefore would not have been beneficial for anabolism. It is likely that the maintained concentrations of essential and branched chain amino acids in the FMP group would sustain amino acid availability to muscle tissue to support anabolism.

Signalling via the mTORC1 pathway is required for MPS to be increased following resistance exercise [19], mTOR exists in a complex with a number of other proteins and its activity is normally measured by the phosphorylation status of its downstream targets [20]. We show that in agreement with previous studies [12, 21, 43] p70S6K^Thr389^ is activated following resistance exercise at both 90 and 240 min after exercise. The phosphorylation of p70S6K^Thr389^ has previously been shown to be sensitive to higher dose protein feeding both at rest and after exercise [43]. Our results suggest that perhaps a larger protein dose would be required to further increase phosphorylation above exercise alone or that an earlier time point may have been required to observe a synergistic protein feeding effect [12]. Although the p70S6K^Thr421/Ser424^ phosphorylation site has been measured less frequently than the p70S6K^Thr389^ site it has been shown to be sensitive to muscle resistance exercise and amino acid intake [44]. While the present study shows that phosphorylation at the p70S6K^Thr421/Ser424^ site is increased by resistance exercise at 90 min post exercise, this response is only sustained at 240 min following the consumption of FMP. rpS6^Ser235/236^ phosphorylation was increased by exercise but maintained at a higher magnitude in the FMP compared to the CHO group. Glover and colleagues report that rpS6^Ser235/236^ phosphorylation was increased by both exercise and a similar dose of protein to the present study but that these increases were not additive [45]. The rpS6^Ser240/244^ site was only phosphorylated by the combination of exercise and protein intake. This site is known to be sensitive to protein intake after resistance exercise [45] but has not previously been tested with the dose of protein or protein source used in the present study. The mitogen-activated protein kinase (MAPK) pathway is also known to converge with mTORC1 signalling to regulate the initiation of protein translation [44]. The MAPK targets ERK1/2 and p90RSK were activated following resistance exercise with protein intake having no effect. Previously it has been shown that the MAPK pathway is primarily sensitive to cellular stress such as that caused by resistance exercise but not responsive to protein feeding [43].

Changes in amino acid transporters were assessed at the level of mRNA and protein expression. In agreement with previous studies, transcriptional changes were more robust than those observed for protein expression [26]. LAT1/ *SLC7A5* gene expression was increased in all conditions at 240 min following beverage consumption. Based on the low concentrations of circulating essential amino acids during both the overnight fast and the study period it is possible that LAT1/*SLC7A5* transcription was increased as a compensatory mechanism in all conditions [46]. The expression of CAT1/*SLC7A1*, PAT1/ *SLC36A1*, and SNAT2/*SLC38A2* mRNA was increased by resistance exercise and unaffected by beverage consumption. This finding is in agreement with work by Drummond et al. [26] that showed resistance exercise mediated increase in amino acid transporter gene expression in both young and older adults, even though, the peak gene expression may have been after the end of the measurement period. Ten grams of essential amino acids have been shown to increase amino acid transporter expression at rest [27], the dose of protein used in the present study equates to ~4.0 grams of essential amino acids. It seems that a dose of essential amino acids larger than the 4.0 grams used in the present study may be required to stimulate amino acid transporter transcription. A synergism between resistance exercise and protein intake for amino acid transporter transcription has never been clearly demonstrated, thus it is unclear if the ingestion of a greater protein dose would have further increased amino acid transporter gene expression after resistance exercise.

The participants in the present study were middle aged. This is unique as most studies that measure muscle anabolism after exercise and feeding investigate either young adults or older adults. For this reason much less is known about the dose response of anabolic signalling to exercise and protein feeding in middle age. It seems reasonable to surmise that the anabolic sensitivity of middle aged men would be intermediate between the high sensitivity of young men [47] and the diminished sensitivity of older men [11, 12]. It is clear that 9 grams of milk protein represents a suboptimal stimulus to promote mTORC1 signalling after resistance exercise. However despite that finding, a product containing 9 to 10 grams of protein could still make the content claim ‘good source of protein’ in many parts of the world [48, 49]. This level of protein intake has been shown to have a detectable anabolic benefit in young men [40, 45] and we extend these findings by showing an anabolic benefit after resistance exercise in middle aged men. The present study was limited by the relatively small sample size and the lack of female participants. Future work should test the effects of small protein does in a larger cohort of both men and women.

## Conclusions

As little as 9 g of high quality milk protein is able to augment the downstream signalling response induced by resistance exercise in middle aged men but does not potentiate the increase in amino acid transporter gene expression after resistance exercise. Therefore, formulated products containing 9 grams of milk protein would be expected to exert a stimulatory effect on muscle anabolism after resistance exercise. This may be of benefit in the development of food products targeted towards population groups that are unable to tolerate single dose high-protein products or that are unfamiliar with the tastes and sensory properties of high-protein supplemental foods and beverages.

## Abbreviations

1RM: One rep maximum
CHO: Carbohydrate
DXA: Dual-energy X-ray absorptiometry
FED: Leg that was exposed nutrition but remained rested
FED-EX: Leg which was exposed to both nutrition and resistance exercise
FMP: Functional milk product
HOMA: Homeostatic model assessment of insulin resistance
MPC: Milk protein concentrate
MPS: Muscle protein synthesis
mTORC1: Mammalian target of rapamycin complex 1
P70S6K: P70S6 kinase
rpS6: Ribosomal protein S6
TBST: Tris buffered saline and tween 20
